# Cases Occurring in the Practice of Prof. D. Brainard

**Published:** 1857-05

**Authors:** Edwin Powell

**Affiliations:** Resident Surgeon in the U.S. Marine Hospital, Chicago, Ill.


ARTICLE IV.—*Cases Occurring in the Practice of Prof. D. Brainard.* Reported by EDWIN POWELL, M.D. Resident Surgeon in U.S. Marine Hospital, Chicago, Ill.

*Case I.*—Rufus Mather, American, æt. thirty-nine; a stout, robust man. Was admitted into U.S. Marine Hospital January 9th, 1857, laboring under retention of urine from stricture.

The patient stated that twelve years previously he fell from a loft in a barn, and struck astride a manger rail, which produced laceration of the perineum and rupture of the urethra, with consequent infiltration of urine.

Abscesses in the perineum, and sloughing of the scrotum followed, which were eventually healed. But a urinary fistula remained, and the urethra is bound down to the os pubis to the left of the symphysis.

From that time to the present, the patient has been afflicted with symptoms of stricture, which, during the last year, have been severe, and for several months the stricture has become more perfect. Once during the interval he has had a perfect retention of urine, but was relieved by the introduction of a catheter. At present the bladder rises to the umbilicus; there is great pain and constant desire with inability to void the urine. The patient has not passed his urine, except *guttatim* for the last seventy-two hours.

On attempting to introduce a catheter, it was found impossible to convey it but a few inches along the urethra before it came in contact with a stricture, beyond which it could not be made to pass. Finding there was no prospect of relieving him in this way, it was decided to incise the stricture by 'perineal section.' The patient, having been brought under the influence of a mixture of chloroform and ether (one part of the former to five of the latter), was placed on the operating table in the position for lithotomy. A grooved sound was passed along the urethra until it came in contact with the stricture—a distance of about six inches—and an incision made down upon the point of it; it was then withdrawn and a director taken from a pocket case was passed through the stricture, upon which a bistoury was placed and the stricture freely incised. A No. twelve catheter was introduced into the bladder and allowed to remain for the first two weeks, being removed and replaced every morning for the purpose of cleansing it. The bladder was washed out with cold water for the first two or three days, at which time all unpleasant symptoms had subsided. The patient progressed favorably from this time forward, until at the end of eight weeks the urine flowed in a full stream from the urethra. The catheter was introduced from time to time to prevent a return of the stricture.

(To be continued.)

